# Structurally Efficient Three-dimensional Metamaterials with Controllable Thermal Expansion

**DOI:** 10.1038/srep34924

**Published:** 2016-10-10

**Authors:** Hang Xu, Damiano Pasini

**Affiliations:** 1 Mechanical Engineering Department, McGill University, 817 Sherbrooke Street West, Montreal, QC, H3A OC3, Canada

## Abstract

The coefficient of thermal expansion (CTE) of architected materials, as opposed to that of conventional solids, can be tuned to zero by intentionally altering the geometry of their structural layout. Existing material architectures, however, achieve CTE tunability only with a sacrifice in structural efficiency, i.e. a drop in both their stiffness to mass ratio and strength to mass ratio. In this work, we elucidate how to resolve the trade-off between CTE tunability and structural efficiency and present a lightweight bi-material architecture that not only is stiffer and stronger than other 3D architected materials, but also has a highly tunable CTE. Via a combination of physical experiments on 3D fabricated prototypes and numeric simulations, we demonstrate how two distinct mechanisms of thermal expansion appearing in a tetrahedron, can be exploited in an Octet lattice to generate a large range of CTE values, including negative, zero, or positive, with no loss in structural efficiency. The novelty and simplicity of the proposed design as well as the ease in fabrication, make this bi-material architecture well-suited for a wide range of applications, including satellite antennas, space optical systems, precision instruments, thermal actuators, and MEMS.

With an increase in temperature, most conventional materials expand with thermo-elastic distortions that can be difficult to accommodate. Materials with low coefficient of thermal expansion (CTE) are less sensitive to temperature changes and thus sought in several areas of engineering, such as precision instruments, scanning electron microscopes, flexible electronics, biomedical sensors, thermal actuators, and MEMS^1^,^2^,^3^. Low CTE materials are also particularly crucial in aerospace components, such as space-based mirrors^3^ and satellite antennas^4^, that are built on earth but operate in outer space where wide temperature swings may cause undesired shape and size shrinkage. Solid materials, such as Invar and other metallic alloys, with intrinsically low or negative thermal expansion exist^5^, and are currently used despite their limitations. One of their drawbacks is the narrow range in which they can operate, which is problematic when large temperature swings, from −150 °C to 150 °C as in outer space, occur. The CTE of ceramics and other brittle solids, on the other hand, is low as well as insensitive to temperature variation^6^,^7^; their brittleness, however, poses challenges because thermal stresses easily peak and lead to abrupt failure. In contrast, the ideal material would have CTE that could be tailored to vanish for the whole range of temperature it operates and would not be brittle, a condition currently unmet by all existing solids. One route to create a material with adjustable CTE is to engineer its structural architecture and purposefully tweak it to yield desired ranges of CTE, from positive to negative values including zero.

Recent works on mechanical metamaterials have shown that the CTE of periodic materials can be tuned by purposefully designing the architecture of their repeating unit and proper selection of the constituent materials^2^,^3^,^8^,^9^,^10^,^11^,^12^,^13^,^14^,^15^,^16^,^17^,^18^,^19^,^20^,^21^,^22^,^23^,^24^,^25^,^26^,^27^,^28^,^29^,^30^,^31^,^32^,^33^,^34^,^35^,^36^. It has been shown that low CTE can be obtained through a purely mechanical, and thus temperature-independent, mechanism^3^, which can be in principle designed to make the material operate over a wide range of temperature. Existing mechanical metamaterials, however, have their limitations. The majority are two dimensional and can accommodate only in-plane thermal deformation^2^,^3^,^8^,^9^,^10^,^11^,^12^,^13^,^14^,^15^,^16^,^17^,^18^,^19^,^20^,^21^,^22^,^23^,^24^,^25^,^28^,^29^,^30^,^34^,^35^. A handful are three-dimensional^9^,^25^,^26^,^27^,^28^,^29^,^30^,^31^,^32^,^33^. Among those, some can generate a large range of CTE, i.e. their CTE tunability is high, but their structural efficiency, i.e. the specific stiffness and strength, is poor, since their architecture deform by bending when loaded^36^, a deformation mechanism that is far from being structurally efficient. A very small set of architectures, on the other hand, are stretch dominated with high specific stiffness, but these concepts can generate only a narrow range of CTE and cannot yield a vanishing CTE. The trade-off between CTE tunability and structural efficiency is currently unresolved with current concepts achieving high CTE tunability only at the expense of a dramatic loss in their specific stiffness and specific strength^25^,^32^, and vice versa^9^,^26^,^27^,^28^,^29^,^30^,^31^,^33^. The goal of this work is to create a structurally efficient 3D architected material with highly tunable CTE including zero.

## Mechanisms of Thermal Deformation in a Tetrahedron

**Figure 1:**
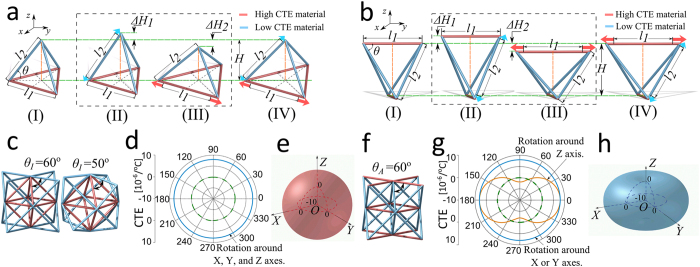
Thermal mechanisms a bi-material tetrahedron featuring either a stationary-node (**a-I**) or stationary-lines (**b-I**). II and III show visually uncoupled thermal expansions, one for the low-CTE material and the other for the high-CTE material; IV shows the overall thermal expansion of the tetrahedron. Application of bi-material tetrahedra to the Octet cells (**c,f**). (**c**) Iso-CTE Octet obtained by assembling 8 stationary-node tetrahedra with skew angle (*θ*_*I*_) of 60° and 50° on a regular octahedron (red core). (**d,e**) isotropic CTE properties plotted in polar and spherical coordinates. (**f**) Aniso-CTE Octet (skew angle *θ*_*A*_ = 60°) with directional CTE obtained with stationary-lines tetrahedra. (**g,h**) anisotropic CTE properties with low CTE in the *z*-direction. The green dashed line in (**d,g**) represents points with zero thermal expansion (Supplementary Information–Video 1).

Since existing architected materials with high CTE tunability are bend-dominated and have thus low specific stiffness and strength, here we start off our investigation by considering a stretch-dominated lattice, the Octet truss, known for its structural efficiency, superior to that offered by most of the other existing lattices. The Octet lattice is obtained by replicating along periodic directions a unit cell that consists of a regular octahedron surrounded on each of its eight faces by a tetrahedron. We first focus on the thermal deformation mechanisms of its main building block, the tetrahedron, and show later how they can be used in an Octet lattice to generate high CTE tunability, equally low in all directions or along preferential directions. A bi-material tetrahedron can undergo two mechanisms of thermal deformation by simply switching the position of the constituent materials. The first mechanism results in stationary-nodes (Fig. 1a)^9^,^28^,^29^,^30^,^31^,^33^,^34^,^35^, and can be used in an Octet cell to generate thermal expansion isotropy. The second mechanism that we identify here for the first time resorts to stationary-lines that appear in a bi-material tetrahedron upon thermal expansion (Fig. 1b). This mechanism can be applied to obtain an Octet lattice with zero CTE in a specific direction, a thermal behavior that is often required at the interface between dissimilar materials, especially in transition components, or adapter elements, in need to tolerate a thermal-expansion mismatch in a given direction^17^. We recall that the unit cell of the Octet truss, here chosen for its high structural efficiency, contain eight tetrahedra that can be used to create thermal expansion mechanisms with either stationary lines or stationary nodes. A systematic strategy that uses the tetrahedron as the building block of the repeating unit is currently developed in a parallel work to rationally design a rich variety of bi-material lattices with programmable thermal expansion.

Figure 1a,b show the thermal expansion mechanisms with a stationary node and stationary lines respectively. The members with higher CTE, *α*_1_, are shown in red and those with lower, but still positive, CTE, *α*_2_, in blue. With a temperature increase, two modes of deformation occur simultaneously in the tetrahedron of Fig. 1a-I, although visualized uncoupled in Fig. 1a-II,III. The blue elements tend to heighten the tetrahedron (Δ*H*_1_ in Fig. 1a-II), whereas its top vertex is retracted by the red elements (Δ*H*_2_ in Fig. 1a-III). If the CTE of the constituent materials, *α*_1_ and α_2_, or alternatively the skew angle, *θ*, are carefully chosen, as shown in Fig. 1b-IV, the height increase of the tetrahedron can be counteracted, a condition that leaves the position of the top vertex unaltered. This point is designated as a stationary-node^35^, which implies that its position in the tetrahedron does not change during thermal deformation (See also Movie S1–Supplementary Information). An alternative scheme of thermal deformation, introduced here, consists of stationary-lines that can appear in a tetrahedron during thermal deformation (Fig. 1b). With this mechanism, the minimum distance (marked as *H* in Fig. 1b) between the top and bottom struts (red) of the tetrahedron, which have identical CTE, remain constant upon heating. For stationary lines to appear, the struts with high CTE (red) should be rationally designed in the tetrahedron so as to be skewed (Fig. 1b), as opposed to those in a stationary-node mechanism, which all lie in the base plane of the tetrahedron (Fig. 1a). In a stationary-line layout, the relative position of the struts, including the angle between struts and the minimum distance (*H*) between the top and bottom struts are preserved upon heating. Similarly to Fig. 1a,1b-II,III show the deformation modes for a stationary-line tetrahedron; here to ease the understanding, the deformations are shown as uncoupled, although in practice they are not, as they occur at the same instant. Figure 1(b-II)) shows the height increase, Δ*H*_1_, caused by the thermal expansion of the blue elements, whereas Fig. 1(b-III) visualizes the height decrease Δ*H*_2_ induced by the greater expansion of the horizontal red beams. The height increase, Δ*H*_1_, of the blue elements (Fig. 1b-II) is thus compensated by a height decrease, Δ*H*_2_, (Fig. 1b-III) induced by the greater expansion of the red members. As a result, the overall CTE of the tetrahedron in the vertical direction (Fig. 1b-IV) can be controlled through either an educated choice of the constituent materials, in particular by selecting the CTE (*α*_1_ and *α*_2_) of the two constituent materials, or a tailored selection of the skew angle, *θ*, between the struts shown in Fig. 1 (See also the Movie S1 in the Supplementary Information).

## Generation of Octet bi-materials with High CTE Tunability

Low CTE tetrahedra with either a stationary-node (Fig. 1a-I) or stationary-lines (Fig. 1b-I) can be used to generate more complex 3D unit cells that in turn can tessellate periodic lattices with low CTE in desired directions. Here we chose three examples as shown in Fig. 1c,f, all applied to an Octet cell but potentially applicable to other cell topologies containing tetrahedra. Figure 1c shows two Octets with a stationary-node, one with skew angle *θ*_I_ = 60° and the other with *θ*_I_ = 50°, and Fig. 1f illustrates an Octet with stationary-lines for *θ*_*A*_ = 60°. The Octet in Fig. 1c, namely Iso-CTE Octet, has a regular octahedron as its core, surrounded by eight stationary-node tetrahedra on its faces. Upon heating, each of the vertices of the eight tetrahedra remains stationary, and can guarantee an isotropic CTE, as shown by the plots in Fig. 1d,e. On the other hand, the Aniso-CTE Octet in Fig. 1f has stationary-lines tetrahedra assembled in an Octet cell that results in thermal expansion anisotropy. In this case, upon heating, the relative position of the planes, where the red struts of the eight tetrahedra lie, remain unaltered, thereby leading to a potentially zero thermal deformation in a specific direction (see Fig. 1g,h). Whereas the thermal expansion of the Iso-CTE Octet is isotropic, its mechanical properties are almost isotropic, as opposed to the Aniso-CTE Octet, which is both mechanically and thermally anisotropic.

## Results

### Manufacturing, simulations, and testing

**Figure 2:**
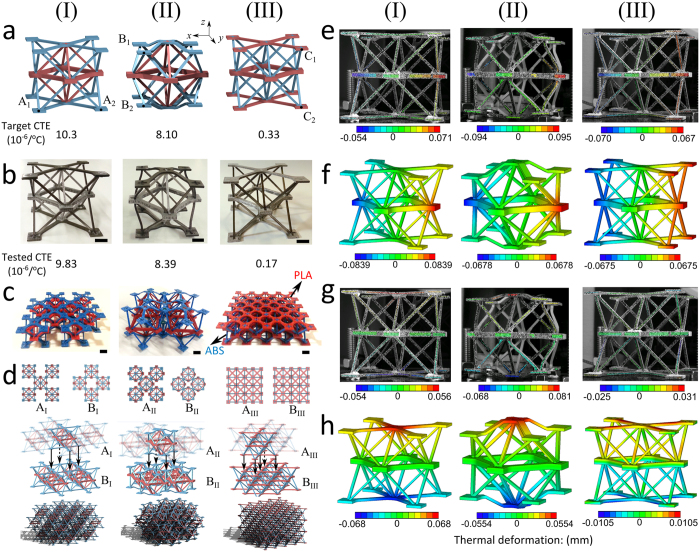
Design embodiments for Iso-CTE Octet and Aniso-CTE Octet: (**a**-I) Topologies for Iso-CTE Octet (*θ*_*I*_ = 60°) with A_1_ and A_2_ used as corresponding nodes for the CTE measurement in the *x*-direction, (**a**-II) Iso-CTE octet (*θ*_*I*_ = 50°) with B_1_ and B_2_ used as corresponding nodes for the CTE measurement in the *z*-direction, and (**a**-III) Aniso-CTE Octet (*θ*_*A*_ = 60°), with C_1_ and C_2_ as corresponding nodes for the CTE measurement in the z-direction; (DIC tested CTEs obtained by evaluating the average of 100 CTE measurements between pairs of corresponding nodes). (**b**) Cells manufactured from Al6061 (high-CTE) and Ti-6Al-4V (low-CTE); Design target and tested CTEs given for Iso-CTE Octet and Aniso-CTE Octet in the *z*-direction. (**c**) 3D printed low-CTE lattices showing one layer of the bi-material lattice: PLA (poly lactic acid) in red with high CTE, and ABS (acrylonitrile butadiene styrene) in blue with low CTE. (**d**) Top and isometric views of two layers showing the stacking sequence as well as the overall lattice assembly for each bi-material concept. Thermal displacement maps for horizontal deformation (**e**: DIC and **f**: FEA) and vertical deformation (**g**: DIC and **h**: FEA). (Scale bar: 10 mm) (Supplementary Information–Movie S2).

The concepts shown in Fig. 1 elucidate the two thermal expansion mechanisms of a tetrahedron and show their application to three Octet cells, where the skew angle can be tuned to yield negative, zero, or positive, CTE in either all directions or a specific direction. We now demonstrate their thermal tunability with physical experiments on fabricated prototypes and examine the robustness of our findings by performing numeric simulations. The embodiment of each Octet cell (Fig. 1) is shown in Fig. 2a with manufactured prototypes in Fig. 2b, each targeting a specific CTE value of design. Each Octet cell is an assembly of laser cut pieces from Al6061 (23 × 10^−6^/°C) and Ti–6Al–4V (11.5 × 10^−6^/°C) alloy sheets with pretension snap-fit joints, similar to simple snap-fit joints^37^. (See Methods Section and Supplementary Information). The thermal properties are tested on a single unit cell with experimentally measured CTE values, reported in Fig. 2b, that enable a quantitative validation of the computational results. The feasibility of the overall lattice assembly is demonstrated in Fig. 2c,d, where geometric models and 3D printed prototypes for each version of the Octet cell show the overall in-plane and out-of-plane tessellation. For CTE measurements, a 3D digital image correlation (DIC) set-up with a temperature controlled heating chamber (Methods Section and Supplementary Information) is used to assess the CTE of each version of the Octet cell. Results from experiments (Fig. 2e,g) and their counterpart simulations (Fig. 2f,h) are quantitatively in good agreement (as further illustrated in Movie S2–Supplementary Information). The results in Fig. 2e-I to 2h-I show that for the Iso-CTE Octet with *θ*_*I*_ = 60°, the thermal expansion along both the *x*- and *z*-direction is low and positive (9.83 × 10^−6^/°C), which is below the CTE value of the inner Al6061 octahedron (21 × 10^−6^/°C), and below that (11.5 × 10^−6^/°C) of its constituent material Ti-6Al-4V. In addition, the effective CTE of the Iso-CTE Octet can be further reduced to 8.39 × 10^−6^/°C by changing the skew angle from *θ*_*I*_ = 60° to 50° (Fig. 2e-II to h-II). On the other hand, for the Aniso-CTE Octet, the CTE measurement in Fig. 2g-III shows no change appears in the vertical distance between the horizontal bars; in addition, the CTE value in the *z*-direction is almost zero (0.171 × 10^−6^/°C), and much lower than the CTE in the horizontal direction (Fig. 2e-III to h-III). We note that the maximum and minimum CTE values obtained with FEA and DIC differ slightly. The reason for the discrepancy is attributed to the testable zone on a given strut where DIC can provide reliable measurements. Points outside this zone, which is smaller than that analyzed with FEA, cannot be measured. Other factors of the DIC technique that can play a role in explaining the difference between experiment and computational results include the size and curvature of the testing sample subset, and the light contrast used during the experiments. (See Methods Section and Supplementary Information).

## Discussion

**Figure 3:**
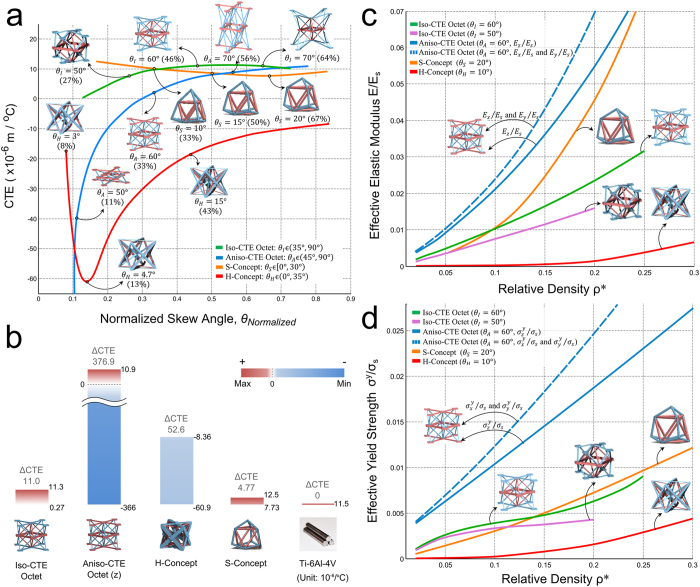
(**a**) Thermal properties of the Octet bi-materials and benchmark concepts (S-concept^9^ and H-concept^32^) plotted with respect to the normalized skew angle, *θ*_*Normalized*_. Shown in bracket below each pictorial, *θ*_*Normalized*_ is calculated as *θ*_*Normalized*_ = (*θ*_*Model*_ − *θ*_*min*_)/(*θ*_*max*_ − *θ*_*min*_), where *θ*_*Model*_ is the skew angle of a concept, and *θ*_*min*_ and *θ*_*max*_ are respectively the minimum and maximum skew angle that a concept can provide without its cell topology degenerates. (**b**) Histogram showing the CTE tunability (∆CTE) as well as the max and min CTE for the selected concepts compared to a low CTE solid material, i.e. Ti-6Al-4V. (**c**) Effective stiffness and (**d**) effective strength plotted with respect to relative density ρ*. Note ρ* = *ρ*_*lattice*_/*ρ*_*solid composite*_ where *ρ*_*lattice*_ is the actual density of the bi-material unit cell and *ρ*_*solid composite*_ is the density of the solid bi-material with volume fraction of the constituent materials. Note that the maximum relative density of the Iso-CTE Octet does not reach 0.3; it is 0.2 for *θ*_*I*_ = 50°, and 0.25 for *θ*_*I*_ = 60°, because above these values the size of the joints become unfeasibly larger than the cell members, which means that the cell is unfeasible.

**Figure 4:**
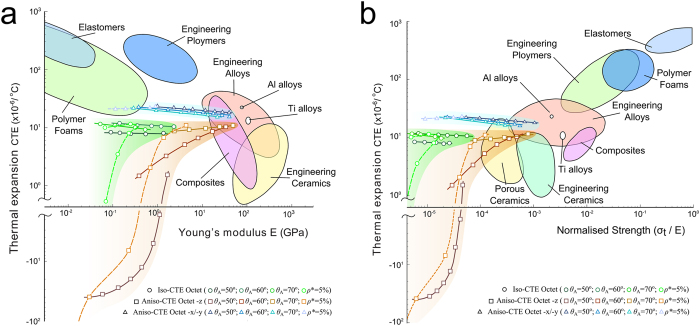
(**a**) CTE plotted against Young’s modulus^39^; and (**b**) CTE versus normalized strength (ultimate strength for Metals and Polymers; modulus of rupture for Ceramics; tensile strength for Composites)^40^. The points for the Octet cells are obtained by varying either the skew angle for a given relative density (0.05) or vice versa. Relative density ranges considered in the charts for the Octet lattice concepts: 0.02 < ρ* < 0.25 for the Iso-CTE Octet, and 0.02 < ρ* < 0.60 for the Aniso-CTE Octet.

To establish and compare the thermomechanical merit of the Octet lattice bi-materials here presented, we now resort to two sets of charts. The first set (Fig. 3) compares the Octet concepts with two 3D benchmarks existing in the literature^9^,^32^ for a representative value of relative density of 0.05 and assuming Al6061 and Ti–6Al–4V as the constituent materials (Material properties in Tables S1 and S2 of Supplementary Information); the second set (Fig. 4) compares them with other classes of materials in a given range of relative density. Figure 3a illustrates the CTE trends with respect to the skew angle for our versions of the Octet, along with those of the two benchmarks, the H-concept^32^ (bend-dominated) and the S-concept^9^ (stretch-dominated). The former has low structural efficiency but high CTE tunability, as opposed to the latter, which is structurally efficient but with relatively poor CTE tunability. CTE tunability is here measured by ∆CTE, which is the difference between the upper and lower CTE limit (obtained from the *y*-axis of Fig. 3a) that each unit cell can yield with a change in either its skew angle or the constituent materials. We recall here that these are two, among others, of the most promising geometric variables that can be controlled to further boost the CTE tunability. The higher the CTE tunability of a concept, the larger ∆CTE. The results obtained for each concept via asymptotic homogenization^38^ and validated via experiments for given geometries, show that the Aniso-CTE Octet has the highest CTE tunability. The CTE in the *z*-direction ranges from −366 × 10^−6^/°C (not visible in Fig. 3a) to 10.9 × 10^−6^/°C, values that result in a tunability ∆CTE_*(θ)*_ = 376.9 × 10^−6^/°C. For larger skew angles, the CTE of the Aniso-CTE Octet monotonically increases to reach that of Ti-6Al-4V. The H-Concept, on the other hand, has a highly tunable CTE, which can also reach negative values; as shown in Fig. 3a; its effective CTE first decreases before rising up again for increasing skew angles. The CTE tunability of the Iso-CTE Octet is more than double that of the S-Concept, which has CTE that reaches the minimum of 0.273 × 10^−6^/°C for *θ*_*I*_ = 42° and its max of 11.3 × 10^−6^/°C for *θ*_*I*_ = 65°. Figure 3b shows a comparison of the CTE tunability of the concepts here under investigation, with those of Ti-6Al-4V. Besides their ∆CTE represented by the height of each column, the red and blue gradients specify CTE ranges of positive and negative values with white indicating zero. The Iso- and Aniso-CTE Octet are the only concepts that can obtain near-zero CTE. The H-Concept can provide a negative CTE range, whereas the ∆CTE of the S-Concept is narrow with min and max values that are both positive. The octet concepts presented in this work have a large CTE tunability including vanishing CTE obtained from common materials, such as Al and Ti alloys, which have both positive CTE.

To assess the structural efficiency of the bi-material Octet lattices, we plot in Fig. 3c,d the effective elastic modulus and yield strength in all the relevant directions for the low range of relative density 0.02 to 0.30. The plot of the shear properties is included in the Supplementary Information. The properties of the H-Concept and the S-Concept are also visualized to allow a comparison of their specific stiffness and strength, represented by the slope of a given point on a curve. We note that for ρ* below 0.1, the relation between the Young’s modulus and the relative density of the bi-material Octet lattice is not linear–as one would expect to be for a single material lattice. The reason for this is that the unit cell contains struts made of two materials with dissimilar relative density. As expected, the results show that all stretch dominated concepts have a better structural efficiency, with the Aniso-CTE Octet being the best for stiffness and strength in the *z*-direction. For relative density above 0.1, the Iso-CTE Octet with *θ*_*I*_ = 60° performs worse than the S-Concept, because the joints of the latter are modelled as ideal, i.e. weightless, as opposed to those of the Octet cells, which are modelled with their actual manufactured geometry. By comparing the two versions of the Iso-CTE Octet, one with *θ*_*I*_ = 50° and the other with 60°, we observe that the former can provide a lower CTE at the expense of a reduction in structural performance. In summary, the Aniso-CTE Octet has the best mechanical performance, whereas the Iso-CTE Octet outperforms the structural performance of the S-Concept and the H-Concept.

We finally resort to material property charts to visualize the thermomechanical performance of the two Al6061-Ti-6Al-4V Octet concepts and compare it with that of other classes of materials. Figure 4a,b show respectively Young’s modulus and normalized strength plotted versus the CTE. The Octets with high CTE tunability achieve unprecedented combinations of properties that cannot be obtained with other classes of materials^39^,^40^. By spanning the skew angle of the Iso-CTE Octet, the CTE can range from near-zero (<1 × 10^−6^/°C) to that of Ti-6Al-4V. The CTE of the Aniso-CTE Octet in the vertical direction can be theoretically changed from negative infinity to the CTE of Ti-6Al-4V. The Aniso-CTE Octet has CTE in the horizontal direction approximately equal to the Al6061 CTE, but possesses Young’s modulus and normalized strength that can be tailored by purposefully changing relative density and skew angle. Figure 4 shows that for a given CTE, the Young’s modulus and normalized strength of the Aniso-CTE Octet in the *z*-direction are higher than those of the Iso-CTE Octet. On the other hand, for given Young’s modulus or normalized strength, the Aniso-CTE Octet can obtain a lower CTE in the *z*-direction, which make it an ideal candidate for applications which require thermal stability in prescribed directions.

## Conclusions

To conclude, this study has presented, built, and thermally tested Octet lattice bi-materials (Al6061-Ti-6Al-4V) that can be tuned to cover a substantial range of CTEs, which include zero, in given spatial directions without jeopardizing their structural efficiency. Several variables defining the unit cell geometry govern the macroscopic thermo-mechanical properties of the bi-material lattices here investigated. The focus of this paper has been on a subset of parameters, i.e. skew angle and strut thickness controlling relative density, which were selected as considered to play a crucial role in the CTE as well as specific stiffness and strength of the lattices. Other factors, such as the strut diameter ratio of the constituent materials and other topological variables, can be studied and used to achieve further CTE tuning. We also note that the conclusions drawn in this work on the thermomechanical performance of the bi-material octet lattice apply to the cases here investigated. Prototypes were built via snap-fit with pretension joints from either tetrahedra with stationary-node to provide CTE isotropy, or tetrahedra with stationary-lines to achieve CTE anisotropy. Results experimentally tested and validated via numeric simulations show that by introducing material architecture even common materials can render unprecedented combinations of thermomechanical properties. The novelty and the simplicity of the proposed 3d design as well as the ease in fabrication, make structurally efficient lattices with tunable coefficient of thermal expansion well-suited for a wide range of applications, such as satellite antennas, space optical systems, space-based mirrors, precision instruments, thermal actuators, and MEMS. The thermal expansion mechanism introduced in this paper for the stationary-line tetrahedron, can also be exploited to create other multifunctional 3d architectures with high structural performance and desired directional properties, including thermal conductivity, acoustic bandgap, and electrical conductivity.

## Methods

### Sample Fabrication

Pretension snap-fits were used to fabricate all the three versions of the Octet cell. Laser cut elements were bended to allow skew angle variation as needed, prior to snap-fitting of diagonal elements. Pretension provided by wedge resistance was used to fasten diagonal elements into cross-shaped holes. The connection between different elements was further reinforced with epoxy glue. The metal fabrication process is summarized schematically in Fig. S1 with further explanatory details given (Supplementary Information).

### DIC Measurements

Digital Image Correlation was used to measure the small deformation of each lattice member and provide a full-field displacement map describing the thermal deformation behavior^3^,^16^,^18^,^35^. A high contrast black and white speckle pattern with proper dot density was painted on each member of the lattice samples. The lattice was then placed into a heating chamber which rose the temperature up to approximately 125 °C starting from an initial temperature of 25 °C (Supplementary Information). The temperature was measured via thermocouples and collected in a data acquisition system. A proportional–integral–derivative controller (PID) was used to control the temperature. Digital images were captured with two charge-coupled-device (CCD) cameras and were controlled by a computer program (Vic-Snap 8^41^). VIC-3D^42^ was used to track the motion of speckle patterns to obtain the thermal deformation. The accuracy of the DIC system was evaluated by tested CTEs from standard metal alloy sheets (Al6061 and Ti-6Al-4V). The displacement noise was evaluated by taking multiple stationary images with time separation of 100 ms. The noise was less than 2 μm, while the expected displacement range was ~100–150 μm.

### Finite Element Simulations and Asymptotic Homogenization

Full 3D models of the testing samples were simulated by performing finite element simulations (ANSYS 14.5^43^). All models were meshed using 10-node tetrahedral elements and simulated under a thermal load of 100 °C. The effective CTEs were calculated by measuring the linear expansion between either the stationary-nodes or the stationary-lines (Fig. 2a). For each cell topology, including the H-Concept and the S-Concept, asymptotic homogenization theory was combined with finite element analysis to calculate the thermomechanical effective properties. For thermal expansion analysis in steady state, the properties including effective stiffness, yield strength, and CTEs in all three orthogonal directions were calculated with respect to the relative density and the skew angle. Thermal properties calculated numerically were further validated for selected samples via experimentally obtained thermal measures. On the role played by the joints in the deformation of the simulated versus manufactured lattices, we recall that the Octet lattice is a pin-jointed structure that is stretch dominated, i.e. its slender members carry axial forces only. Making the joints of the Octet rigid makes no difference in the deformation modes. Whereas the computational analysis has considered both types of joints (pin and rigid), the manufactured lattices are built with rigid joints that are snap-fit to allow proper connection between struts of dissimilar materials.

## Additional Information

**How to cite this article**: Xu, H. and Pasini, D. Structurally Efficient Three-dimensional Metamaterials with Controllable Thermal Expansion. *Sci. Rep*. **6**, 34924; doi: 10.1038/srep34924 (2016).

## Supplementary Material

Supplementary Information

Supplementary Movie S1

Supplementary Movie S2
