# Supplementary material for: Structurally Efficient Three-dimensional Metamaterials with Controllable Thermal Expansion
Source: Sci Rep. 2016 Oct 10;6:34924. doi: 10.1038/srep34924 (PMC5056354; doi:10.1038/srep34924)
Supplement: Supplementary Information [file srep34924-s1.pdf]

Supplementary Information

Structurally Efficient Three-dimensional Metamaterials with Controllable Thermal Expansion

Hang Xu and Damiano Pasini*

S.1. Manufacturing: Pretension Snap-Fit Method

Figure S1 shows the pretension snap-fit technique used to build the bi-material Octet cell. First, the structural elements were laser cut from metal sheets. For the regular Octet with either θ_I =60° or θ_A =60°, inclined elements were snap-fitted without bending and then attached to the horizontal panels. For irregular Octet-trusses, both the beam elements and interlayer panels require bending. To avoid rebound deformation, sheet metal hot extrusion processes can be used to alter the skewness angle. Since the snap-fit connections are between Al6061 (high CTE) and Ti-6Al-4V (low CTE), when heated, the cross-shaped grooves on the aluminum panels have a larger expansion than the titanium inserts, which loosens the joints, weakens the low CTE mechanism, and might cause the whole structure to fall apart. To avoid joint loosening, wedges with steep slopes (15°) were designed on both skewed elements and horizontal panels. When inserted, the wedged forces provided by the oblique-wedge clamping constrained the joints. Moreover, high-strength high-temperature-resistant epoxy glue (LePage Gel Epoxy Syringe Glue with working temperature up to 150°C) was used to fill in and cement Al6061 and Ti-6Al-4V elements. Epoxy providing a much larger CTE ($45\text{-}65\times10^{-6}$/°C compared with Al6061 and Ti-6Al-4V) can increase the fastening force and prevent the joints from loosening. The wedges can be either laser cut or fine finished by CNC. We emphasize that joints made of Al6061 and Ti-6Al-4V can also be vacuum brazed with TiBrazeAl-580 foil (Al-12Si foil and Cu foil, Titanium Brazing, Inc.).

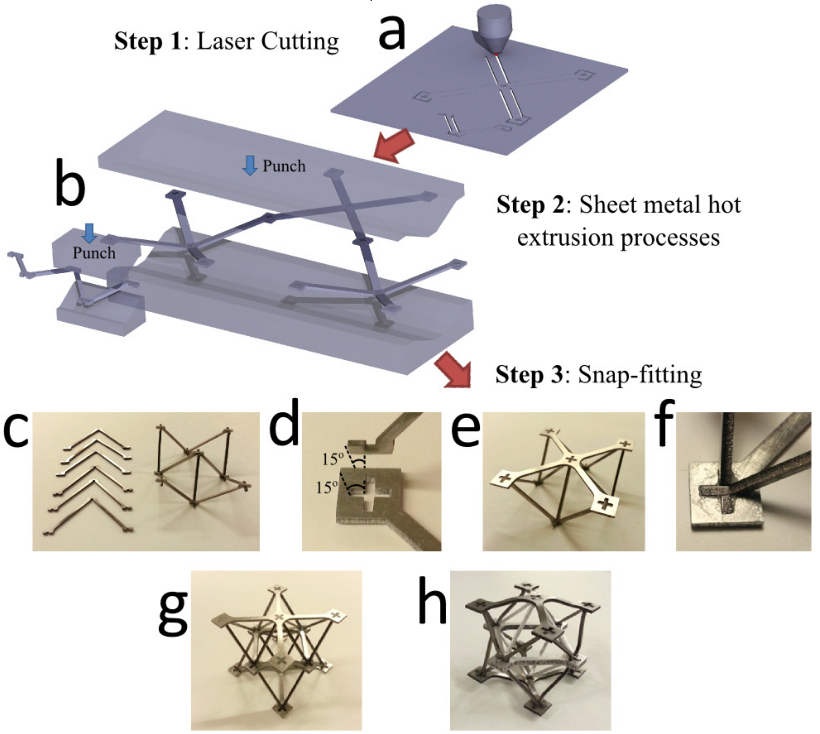

Figure S1: Pretension snap-fit manufacturing process. (a) Laser cutting; (b) Sheet metal hot extrusion processes to impose a given skew angle; (c) Snap-fitting of diagonal elements; (d) Wedge for pretension; (e) Snap-fitting

of horizontal elements; (f) Joint reinforcement via Epoxy Glue; (g) Octet cell assembly from unbended elements; and (h) Octet cell assembly from slightly bended elements.

S.2. Assembly and tessellation rules

Figure S2 shows possible tessellations for Octet cells at given skew angles. A packing density of 100% could be obtained for all Aniso-CTE Octets (Figure S2 a). In contrast, dissimilar skew angles for the Iso-CTE Octet result in tessellations with lower packing factors, i.e. 50% for concepts in Figure S2 b, c, and d, and 100% for the concept in Figure S2 e. Differences in the packing factor are controlled by the need of the inner octahedron to thermally expand without touching adjacent cells as shown in Figure S2 c-II.

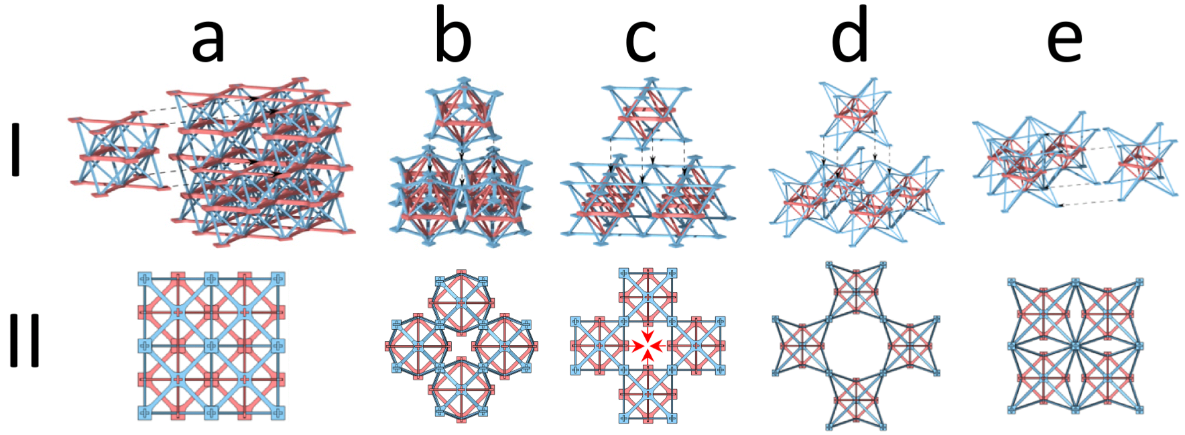

Figure S2: Tessellations of bi-material Octet cells (a) Aniso-CTE Octet with θ_A=60°, (b) Iso-CTE Octet with θ_I=50°, (c) Iso-CTE Octet with θ_I=60°, (d) and (e) are Iso-CTE Octet with θ_I=70°. Assembly in axonometric view (I) and top view (II).

S.3. Experiment to test thermal expansion

Figure S3 shows the schematic of the experimental set-up built to test the thermal expansion of the Octet cells. It consists of a heating chamber and a DIC system. The former with dimensions of 200(L)×200(W)×150(H) mm is made of glass to provide an unobstructed view for DIC measurements. A 200 Watt strip heater was placed under the copper plate in the heating chamber. The copper plate is supported by four leveling mounts which were used for horizontal adjustment. The temperature was controlled by a feedback system, which included a K-type thermocouple attached to the copper plate and a proportion integration differentiation (PID) (OMEGA CN7800) temperature controller. The controllable temperature range was from 25°C to 200°C. The temperature-time data in the PID controller were imported through a RS-485 interface. A NI cDAQ 9174 data acquisition system was used to collect the other three thermocouples from different locations in the chamber and to measure the temperature heterogeneity. A fan in the chamber mixed the air to provide a uniform ambient temperature. The temperature inhomogeneity was regulated within 5% of the real-time temperature through the application of a rotational air fan. Testing samples were covered by a black and white pattern and were set on the copper plate with thermal transfer grease between the two. The thermal strain was measured by DIC using images that were captured through two CCD cameras (Grasshopper 5.0 MP with Fujinon HF25SA-1 lens). Using the DIC correlation software, Vic-3D (Correlate Solution Inc.), virtual extensometers were placed on the reference image and tracked through the images to measure the displacement between pairs of pixel subsets. The strain field was obtained from the

relative displacement between these pairs of subsets. The average temperature CTE was calculated from thermal strain and temperature change.

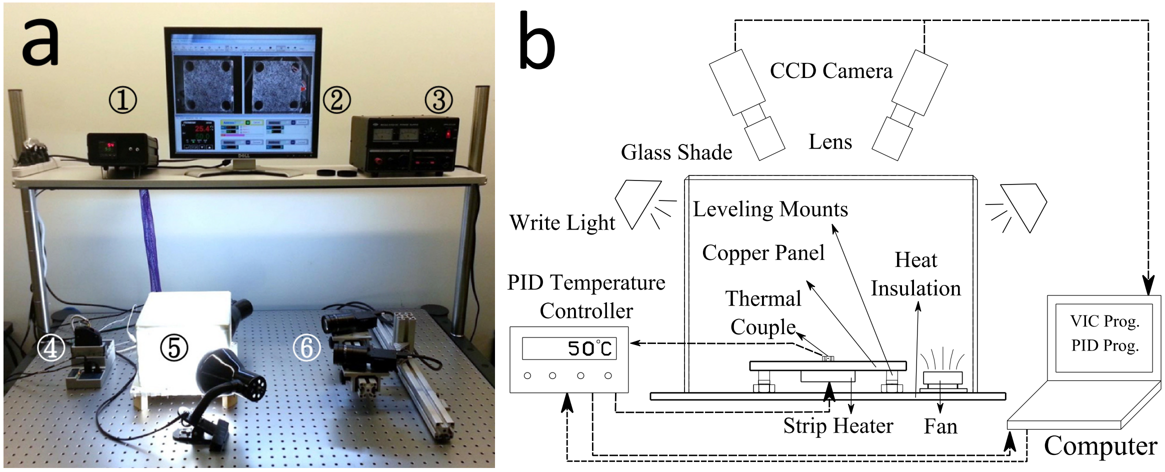

Figure S3: Experimental set-up used to measure thermal expansion (photograph (a) (1 PID controller; 2 Computer; 3 Power source; 4 DAQ system; 5 Heating chamber; and 6 CCD cameras) and schematic picture (b)).

To evaluate the accuracy of the CTE testing system, we choose Al6061 (Ta Chen International Inc.) and Ti-6Al-4V (RTI International Metals, Inc.) as two standard materials. The CTEs of these two standard materials were tested by both our DIC experimental set-up and a commercial thermal expansion instrument (TA Q400, TA Instruments, Inc.). The comparison of the measured CTEs shown in Table S1 shows the accuracy of our experimental set-up.

Table S1: The measured CTEs of Al6061 and Ti-6Al-4V by DIC and TA Q400

Material	DIC	Commercial instrument
Al6061	$22.6\times10^{-6}/°C$	$23.0\times10^{-6}/°C$
Ti-6Al-4V	$12.0\times10^{-6}/°C$	$11.5\times10^{-6}/°C$

Table S2: The mechanical properties of Al6061 and Ti-6Al-4V

Material	Young’s Modulus	Poisson’s Ratio
Al6061	70.75Gpa	0.35
Ti-6Al-4V	113.8GPa	0.34

S.4. Shear Modulus of Low-CTE Octet Truss

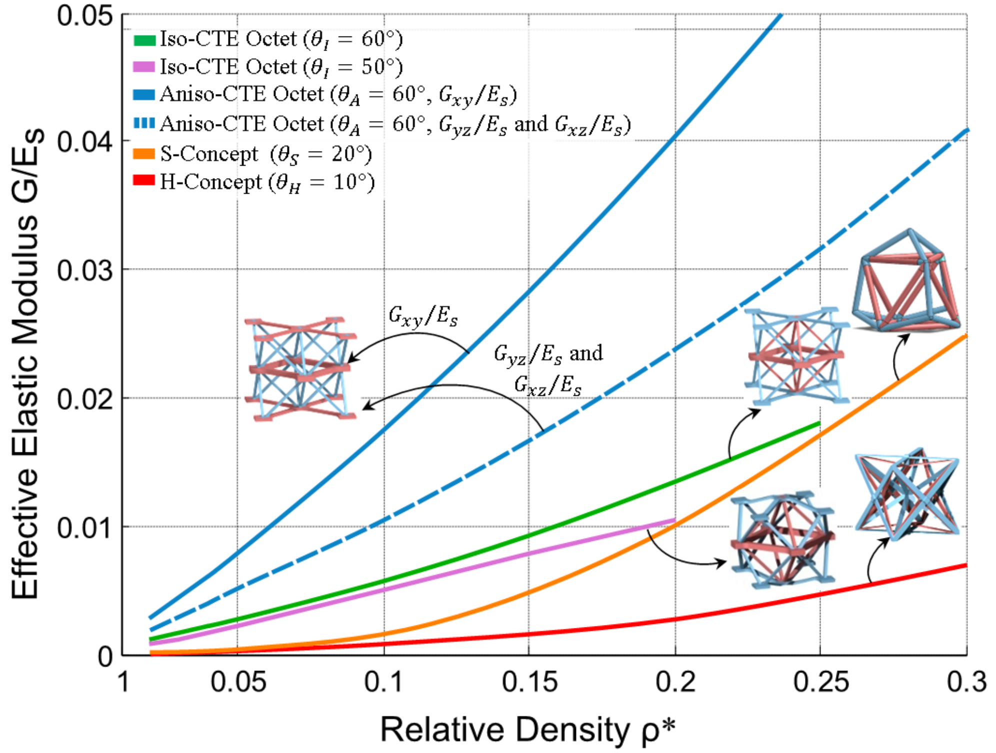

Figure S4: Effective shear modulus plotted with respect to relative density ρ^*

Movie description

Movie S1 shows the thermal expansion displacement of the tetrahedra with stationary-nodes and stationary-lines, and Movie S2 shows the tested and simulated displacement induced by the thermal expansion for the Iso-CTE Octet.
